# Oxidative Stress and Pro-Inflammatory Status in Patients with Non-Alcoholic Fatty Liver Disease

**DOI:** 10.3390/antiox9080759

**Published:** 2020-08-16

**Authors:** Margalida Monserrat-Mesquida, Magdalena Quetglas-Llabrés, Manuela Abbate, Sofía Montemayor, Catalina M. Mascaró, Miguel Casares, Silvia Tejada, Itziar Abete, Maria Angeles Zulet, Josep A. Tur, J. Alfredo Martínez, Antoni Sureda

**Affiliations:** 1 Research Group in Community Nutrition and Oxidative Stress, University of the Balearic Islands and Health Research Institute of Balearic Islands (IdISBa), 07122 Palma de Mallorca, Spain; margalida.monserrat@uib.es (M.M.-M.); m.quetglas@uib.es (M.Q.-L.); manuela.abbate@uib.es (M.A.); sofiamf16@gmail.com (S.M.); catalinamaria95@hotmail.es (C.M.M.); silvia.tejada@uib.es (S.T.); antoni.sureda@uib.es (A.S.); 2 CIBER Fisiopatología de la Obesidad y Nutrición (CIBEROBN), Instituto de Salud Carlos III (ISCIII), 28029 Madrid, Spain; iabetego@unav.es (I.A.); mazulet@unav.es (M.A.Z.); jalfredo.martinez@imdea.org (J.A.M.); 3 Health Research Institute of Balearic Islands (IdISBa), 07120 Palma de Mallorca, Spain; 4 Radiodiagnosis Service, Red Asistencial Juaneda, 07011 Palma de Mallorca, Spain; casaresmiguel@gmail.com; 5 Laboratory of Neurophysiology, University of the Balearic Islands, 07122 Palma de Mallorca, Spain; 6 Department of Nutrition, Food Sciences, and Physiology, Center for Nutrition Research, University of Navarra, 31008 Pamplona, Spain; 7 Cardiometabolics Precision Nutrition Program, IMDEA Food, CEI UAM-CSIC, 28049 Madrid, Spain

**Keywords:** nonalcoholic fatty liver disease (NAFLD), oxidative stress, inflammation, cytokine, steatosis, liver

## Abstract

Background: Nonalcoholic fatty liver disease (NAFLD) is characterized by excessive fat accumulation, especially triglycerides, in hepatocytes. If the pathology is not properly treated, it can progress to nonalcoholic steatohepatitis (NASH) and continue to fibrosis, cirrhosis or hepatocarcinoma. Objective: The aim of the current research was to identify the plasma biomarkers of liver damage, oxidative stress and inflammation that facilitate the early diagnosis of the disease and control its progression. Methods: Antioxidant and inflammatory biomarkers were measured in the plasma of patients diagnosed with NAFLD (*n* = 100 adults; 40–60 years old) living in the Balearic Islands, Spain. Patients were classified according to the intrahepatic fat content (IFC) measured by magnetic resonance imaging (MRI). Results: Circulating glucose, glycosylated haemoglobin, triglycerides, low-density lipoprotein-cholesterol, aspartate aminotransferase and alanine aminotransferase were higher in patients with an IFC ≥ 2 of NAFLD in comparison to patients with an IFC of 0 and 1. The plasma levels of catalase, irisin, interleukin-6, malondialdehyde, and cytokeratin 18 were higher in stage ≥2 subjects, whereas the resolvin D1 levels were lower. No differences were observed in xanthine oxidase, myeloperoxidase, protein carbonyl and fibroblast growth factor 21 depending on liver status. Conclusion: The current available data show that the severity of NAFLD is associated with an increase in oxidative stress and proinflammatory status. It may be also useful as diagnostic purpose in clinical practice.

## 1. Introduction

The most common chronic liver disease in western societies is nonalcoholic fatty liver disease (NAFLD), which affects up to 25% of the population and it is emerging as a serious and growing clinical problem with a 90% prevalence among obese individuals [1]. The prevalence of the more progressive form of NAFLD, nonalcoholic steatohepatitis (NASH), ranges from approximately 25–70% among obese patients [2]. NAFLD is characterized by an excessive fat accumulation, especially triglycerides in hepatocytes, and, consequently, is strongly linked to overweight, obesity, and insulin resistance [3]. NAFLD is accompanied by a broad spectrum of clinical and pathological manifestations hardly distinguishable from those seen in alcoholic patients [3]. If this pathological situation is not properly treated, it can progress to NASH and continue to fibrosis, cirrhosis or even hepatocarcinoma [4]. This disease is not directly associated with age since it can affect people younger than 40 years old [5]. NAFLD could be an additional risk factor for cardiovascular disease (CVD), chronic kidney disease, endocrinopathies (including type 2 diabetes mellitus (T2DM) and thyroid dysfunction) and osteoporosis [6,7,8,9]. NAFLD is a disease with no well defined signs or symptoms, and these include an enlarged liver, fatigue, pain in the right upper abdomen, and a slight increase in circulating transaminases [10]. To date, there are no effective pharmacological therapies against NAFLD, but therapeutic approaches to fight against this disease are basically dietary and lifestyle modifications [11]. Concretely, exercise and nutritional interventions are the first line of therapy, which are mainly aimed at controlling body weight, metabolic syndrome and cardio-metabolic risk factors [12].

Nowadays, the most reliable method of diagnosing NAFLD is through a liver biopsy, but since it is a long-term disease and an invasive method, it is complex to follow large groups of people through serial biopsies [13]. Other methods for diagnosis include a complete ultrasound, which is usually the first test when liver disease is suspected, magnetic resonance imaging (MRI), which allows for a good diagnosis, and elastography, which is an improved form of ultrasound to measure liver stiffness, indicative of fibrosis or scarring [14]. Therefore, many people suffering from NAFLD are not diagnosed until the disease has progressed to a more serious stage. In fact, in a significant number of cases, the diagnosis is made when there is already severe liver disease or cirrhosis and the patient may require a liver transplant [15]. Indeed, about 20–25% of adults with NAFLD develop cirrhosis in 10 years and 11.3% of cirrhotic patients with NAFLD develop hepatocellular carcinoma in 5 years [11].

Oxidative stress and inflammation are significant features involved in NAFLD. Reactive oxygen species (ROS) overproduction can initiate lipid peroxidation processes by damaging both the membrane structure and function, and may be responsible for the oxidation of key proteins for cell metabolism and function, and may cause nucleic acid oxidation [16,17]. All these actions can trigger apoptotic processes by affecting the mechanisms involved in the regulation of the cell life cycle [18,19,20]. Since the liver has a limited ability for triglyceride accumulation, lipid deposition under overfeeding conditions, as in the case of NAFLD, determines the accumulation of high levels of fatty acids, generally saturated ones, which are associated with cell dysfunction [21]. Indeed, the excess of fatty acids induces high rates of β-oxidation, increasing ROS production in the mitochondrial respiratory chain, which can cause cellular damage, and oxidative stress [22]. This situation is directly associated with an increase in oxidative damage markers, an activation of Kupffer cells and pro-inflammatory pathways, and the recruitment of circulating immune cells [23,24]. Chronic inflammation derives from an incorrect resolution of the acute inflammation, which can occur when the source stimulus persists over time. The most common cause of this pro-inflammatory condition is often associated with metabolic diseases, such as diabetes, obesity, metabolic syndrome, nonalcoholic fatty liver, and even in cancers which are characterized by a subclinical chronic inflammatory state [25]. The presence of NASH-associated inflammation identifies NAFLD patients at a higher risk of fibrosis and disease progression [26].

Since the diagnosis of this pathology needs invasive or expensive methods, it is important to find additional markers that allow for the evaluation of the degree of liver steatosis. The aim of this study was to identify the plasma biomarkers of liver damage, oxidative stress and inflammation that facilitate an early diagnosis of the disease and control its progression.

## 2. Materials and Methods

### 2.1. Design and Participants

One hundred 40–60 year-old adults recruited in the Balearic Islands, Spain, were selected considering the following inclusion criteria: (1) BMI (body mass index) 27–30 Kg/m^2^ or an increased waist circumference of ≥94 cm in men and ≥80 cm in women; (2) triglycerides levels ≥150 mg/dL; (3) reduced HDL-cholesterol <40 mg/dL in men and <50 mg/dL in women; (4) increased blood pressure (BP), systolic BP ≥ 130 mmHg or diastolic BP ≥ 85 mmHg; (5) fasting serum glucose level ≥100 mg/dL. The following exclusion criteria were applied: previous cardiovascular disease; liver diseases (other than NAFLD); viral, autoimmune and genetic causes of liver disease; active cancer or a history of malignancy in the previous 5 years; previous bariatric surgery; nonmedicated depression or anxiety; alcohol (>21 and >14 units of alcohol a week for men and women, respectively) and drug abuse; pregnancy; primary endocrinological diseases (other than hypothyroidism); weight loss medications in past 6 months; concomitant therapy with steroids; inability or unwillingness to give informed consent or communicate with study staff.

The study protocols followed the Declaration of Helsinki ethical standards and all the procedures were approved from the Ethics Committee of the Balearic Islands (ref. IB 2251/14 PI). All participants were informed of the purpose and the implications of the study, and all provided the written consent to participate. This study has been registered in Clinicals Trials.gov ref. NCT04442620 [27].

### 2.2. General Data

Information on patients’ socioeconomic status, medical history, and current use of drugs, previous diseases, smoking status and alcohol consumption were obtained from all patients during an initial interview with the study dietician and study nurse.

### 2.3. Diagnosis of NAFLD

The fatty liver analysis was performed with a 1.5-T Magnetic Resonance Imaging (MRI) (Signa Explorer 1.5T, General Electric Healthcare, Chicago, IL, USA) by using a 12-channel phased-array coil [28].

The upper abdominal MRI imaging protocol included the IDEAL IQ sequence, which provides volumetric whole-liver coverage in a single breath-hold and generates estimated T2* and triglyceride fat fraction maps in a noninvasive manner [29]. Breath-held abdominal imaging is able to evaluate diffuse liver diseases such as hepatic steatosis of the liver and corrects for challenging confounding factors such as T2* decay. The technique is designed for water-triglyceride fat separation with a simultaneous T2* correction and estimation based on the IDEAL technique. Six gradient echoes are typically collected using the 3D Fast SPGR sequence in one or two repetitions. The IDEAL IQ reconstruction produces water and triglyceride fat images, and a relative triglyceride fat fraction and R2* maps from the six echo source data.

The patients who met the inclusion criteria and agreed to participate in the study were classified into three groups according to the intrahepatic fat content (IFC) after performing MRI. The first group included patients without evidence of NAFLD (IFC 0); the second group included patients with IFC 1 of NAFLD and the third group included patients with IFC 2–3 of NAFLD. Cross-validated estimates of the diagnostic accuracy of MRI according to the liver proton density fat fraction (PDFF) threshold for grading hepatic steatosis were: IFC 0 (<6.4%), IFC 1 (6.4–17.39%), IFC 2–3 (>17.4%) [30]. Patients were classified as “IFC 0” (*n* = 44), “IFC 1” (*n* = 40) and “IFC ≥2” (*n* = 27) as described above and following the recognized clinical criteria [31,32].

### 2.4. Anthropometric Characterization

Weight (kg) was measured with calibrate scales and the subjects in bare feet and light clothes, so 0.6 kg was subtracted for their clothing. Height (m) was determined to the nearest millimetre with a wall-mounted stadiometer (Seca 213, SECA Deutschland, Hamburg, Germany) with the participant’s head in the Frankfurt plane. Body mass index (BMI) was calculated according to kg/m^2^. Blood pressure was measured in triplicate in a seated position with a validated semi-automatic oscillometer (Omron HEM, 750CP, Hoofddorp, The Netherlands).

### 2.5. Blood Collection and Analysis

After 12-h overnight fasting conditions, venous blood samples from the antecubital vein were collected in suitable vacutainers with ethylenediaminetetraacetic acid (EDTA) anticoagulant, and plasma obtained by centrifuging whole fresh blood at 1700× *g* 15 min at 4 °C. Biochemical parameters: glycosylated haemoglobin (Hb1Ac), total cholesterol, high-density lipoprotein-cholesterol (HDL-c), low-density lipoprotein (LDL) and triglycerides (TG), bilirubin, aspartate aminotransferase (AST), alanine aminotransferase (ALT), gamma-glutamyl transferase (GGT) and c-reactive protein (CRP) were determined using standardized clinical procedures. The hematological parameters (hematocrit) and cell counts (erythrocytes, leukocytes, and platelets) were analyzed in whole blood (automatic flow cytometer analyzer Technion H2, Bayer, VCS system).

### 2.6. Protein Carbonyl Determination

Protein carbonyl derivatives were measured by using an OxiSelect^TM^ Protein Carbonyl Immunoblot Kit (CELL BIOLABS^®^, San Jose, CA, USA) following the manufacturer’s instructions. The total protein concentration was determined by the Bradford method [33] using the Sigma-Aldrich Bradford reagent (Sigma-Aldrich, St. Louis, MO, USA). Firstly, 10 μg of plasma protein was transferred onto a nitrocellulose membrane by the dot blot method (Bio-Rad, CA, USA). Then, the membrane was incubated in carbonyl determination with 2,4-dinitrophenylhydrazine (DNPH). This step was followed by incubation with the primary antibody, specific to DNPH (1:1000). After that, the membrane was incubated with goat antirabbit IgG (1:1000). An enhanced chemiluminescence kit (Immun-Star^®^ Western C^®^ Kit reagent, Bio-Rad Laboratories, Hercules, CA, USA) allows for the development of immunoblots. An image analysis program, Quantity One (Bio-Rad Laboratories, CA, USA), was used to visualize and quantify the protein carbonyl bands.

### 2.7. Enzymatic Determinations

The activities of catalase (CAT) and superoxide dismutase (SOD) were determined both in plasma as described elsewhere [34,35]. Both enzyme activities were measured with a Shimadzu UV-2100 spectrophotometer (Shimadzu Corporation, Kyoto, Japan) at 37 °C. Plasma CAT activity was measured using Aebi’s spectrophotometric method based on the decomposition of H_2_O_2_ [34]. Plasma SOD activity was measured by an adaptation of McCord and Fridovish’s method [35].

### 2.8. Malondialdehyde Assay

A marker of lipid peroxidation in plasma (malondialdehyde; MDA) was measured using the specific colorimetric assay kit (Sigma-Aldrich Marck^®^, St. Louis, MO, USA), whose method is based on the reaction of MDA with a chromogenic reagent generating a stable chromophore. Plasma samples and standards were placed in glass tubes containing n-methyl-2-phenylindole in acetonitrile:methanol (3:1); HCl (12 N) was then added, and the samples were incubated at 45 °C/1 h, and the absorbance was measured at 586 nm. A standard curve of known concentrations was used to calculate the MDA concentration.

### 2.9. Immunoassay Kits

Myeloperoxidase (MPO) and xanthine oxidase (XOD) levels were measured in plasma using ELISA kits following the supplies guidelines for use (Cusabio^®^ Technology Llc, Houston, TX, USA). Irisin levels were measured in plasma using an ELISA kit (Cell Biolabs^®^, San Jose, CA, USA). Resolvin D1 (RvD1) was determined in plasma using an ELISA kit (CaymanChemical^®^, Ann Arbor, MI, USA). Interleukin 6 (IL-6) and fibroblast growth factor 21 (FGF21) were determined in plasma using individual ELISA kits (Elabscience^®^, Houston, TX, USA). Cytokeratin 18 (CK-18) levels were estimated using the M30 Apoptoense^®^ ELISA and measured in plasma following the manufacture’s instructions (PEVIVA^®^, in USA, Canada, and Japan).

### 2.10. Statistics

Statistical Package for Social Sciences (SPSS v.25 for Windows, IBM Software Group, Chicago, IL, USA) was used to carry out the statistical analysis. Results were expressed the mean ± standard error of the mean (SEM). The level of significance was considered at *p* < 0.05 for all statistics. A Kolmogorov–Smirnov test was previously applied to assess the correct distribution of the data. The statistical significance of the data was assessed by a one-way analysis of variance (ANOVA). A Bonferroni post-hoc test was used in order to make multiple comparisons. The biomarker results were analysed by a receiver operating characteristic (ROC) curve and area under the curve (AUC), and by a multivariate logistic regression acccording to intrahepatic fat content IFC (dependent variable) after adjustments for sex, smoking, and alcohol consumption (categorical variables), and age (continuous variable) to control for potential confounding.

## 3. Results

### 3.1. Anthropometric and Haematological Parameters

The anthropometric characteristics of participants stratified by IFC are shown in Table 1. An IFC ≥ 2 showed significantly higher values in weight, glucose, Hb1Ac, TG, AST and ALT with respect to IFC = 0. The systolic blood pressure and ALT also evidenced significant differences when compared with IFC = 1. The HDL-cholesterol was lower when IFC ≥ 2 than stage 0. LDL-cholesterol also showed significant differences. No differences were reported in the haematological variables of the participants, except leukocytes, which was significantly different when IFC = 1 when compared with IFC = 0.

### 3.2. Oxidative Stress and Inflammatory Biomarkers

The results of the enzymatic activities of CAT and SOD, and the ELISA assay of MPO, XOD, irisin, IL-6, and resolvin D1 and biomarkers of plasma damage such as MDA and protein carbonyl are shown in Table 2. CAT, SOD and irisin were significantly higher in subjects with IFC ≥ 2 of NAFLD than IFC = 0 and 1, while no differences were found in MPO, XOD, and protein carbonyls between groups. Differences between participants when IFC ≥ 2 were found for IL-6 which was significantly higher than IFC 0. The RvD1 reported differences between IFC = 0 and IFC ≥ 2, with significantly lower levels when IFC ≥ 2. As a lipid peroxidation marker, MDA levels were significantly higher when IFC = 1 and IFC ≥ 2 compared to when IFC = 0. Differences between sex were observed in CAT (IFC = 1 and IFC ≥ 2), IL-6 (UFC = 0) and MDA (IFC = 0 and IFC ≥ 2).

### 3.3. CK-18 and FGF21 Levels

**Figure 1:**
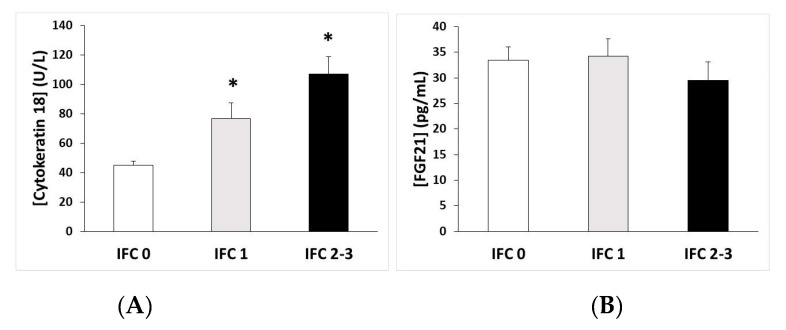
(**A**) Cytokeratin-18 and (**B**) fibroblast growth factor 21 (FGF21) plasma levels (mean ± SEM) according to the intrahepatic fat content (IFC). * *p* < 0.05 respect to IFC = 0 by a one-way ANOVA.

The plasma levels of the CK-18 and FGF21 stratified by the NAFLD stages are shown in Figure 1. The levels of CK-18 were significantly higher when IFC = 1 and when IFC ≥ 2, with respect to when IFC = 0 (Figure 1A), whereas the levels of FGF21 showed no differences (Figure 1B).

### 3.4. ROC Curve of Biomarkers According to IFC

**Figure 2:**
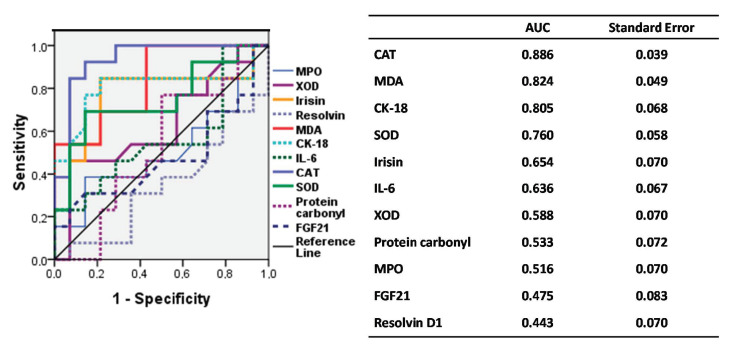
ROC curve of the accuracy of biomarkers in the assessment of the intrahepatic fat content. AUC: Area under the curve; CAT, Catalase; MDA, Malondialdehyde; CK-18: Cytokeratin 18; SOD, Superoxide dismutase; IL-6, Interleukin 6; XOD, Xanthine oxidase; MPO, Myeloperoxidase; FGF21: Fibroblast Growth Factor 21.

Figure 2 shows the accuracy of biomarkers in the assessment of IFC by means of a ROC curve (IFC pathologic or IFC ≥ 1 vs. IFC nonpathologic or IFC = 0). The best area under the curve (AUC) results were found for CAT, MDA, CK-18, and SOD, irisin, and IL-6, representing 88%, 82%, 80%, 76%, 65% and 63% of the AUC, respectively, all over the reference line. Resolvin D1 is a bad predictor according to the AUC values (44%); however, its inverted value could be a good IFC predictor.

### 3.5. Association of Biomarkers and Intrahepatic Fat Content (IFC)

Table 3 shows the association of biomarkers and IFC, by means of a multivariate adjusted logistic regression (odds ratio and 95% CI) considering nonpathological (IFC = 0) as a reference value. After adjusting for possible confounders, catalase, malondialdehyde, cytokeratin 18; SOD, superoxide dismutase; IL-6, irisin, interleukin 6, and the inverse of resolvin was significantly associated to pathological IFC (IFC ≥ 1).

## 4. Discussion

The main findings of this study are that oxidative stress and proinflammatory biomarkers are clearly related to the intrahepatic fat content, which may be useful for diagnostic purposes in clinical practice.

Moreover, the current findings also confirmed previous results [36,37,38] on blood biochemical markers (higher glycaemia, Hb1Ac, triglycerides, AST, and ALT, and lower HDL-cholesterol), which progressively get worse according to the IFC, as well as the altered levels of systolic and diastolic blood pressure. These outcomes are in accordance with previous studies that evidenced significant increases in the specific liver enzymes (ALT, GGT, and AST/ALT ratio <1) and in Hb1Ac in NAFLD subjects as compared to healthy subjects [36,37,38]. It has also been suggested that bilirubin and CRP could be good biomarkers for a good prediction of NAFLD [38,39,40]. The absence of differences in the levels of bilirubin, GGT, and PCR in the present study could derive from the fact that all the patients suffered from metabolic syndrome and there are no healthy patients.

Participants with a high IFC showed higher levels of oxidative damage markers (MDA), plasma antioxidant enzymatic activities (CAT, SOD), proinflammatory markers (IL-6, CK-18), and cytokines (irisin), but lower resolvin D1 levels, and no changes in protein hepatokynes (FGFD21). Previous studies showed increases in the antioxidant enzymatic levels in serum/plasma samples [37] as an adaptive mechanism to cope with the increase in ROS production associated with NAFLD [41]. This higher production of ROS, mainly derived from the respiratory chain [42], can cause cell damage, activate inflammatory cells, and induce cytokine production [37,43]. The accumulation of lipids in liver cells also induces lipotoxicity associated with endoplasmic reticulum stress [44,45], contributing to the induction of inflammatory responses and the development of chronic metabolic diseases such as NAFLD [46]. The current findings are in accordance with previous results, which showed high levels of MDA in NAFLD and chronic viral hepatitis patients [47].

The MPO and XOD levels, as biomarkers of pro-inflammatory states, did not evidence significant differences between their IFCs. Previous studies suggested that MPO could be a good noninvasive biomarker to distinguish NASH from steatosis [48,49]. XOD is an essential enzyme in the metabolism of nucleic acids, which is released into the circulation when liver damage occurs [50]; it mediates the peroxidation of lipids, and is involved in the occurrence and progression of liver damage [51,52]. The absence of differences may be because the participants in the current study have a high BMI and a low-grade subclinical inflammatory state, but they do not have additional inflammation associated with steatohepatitis. To our knowledge, there are no previous studies analysing plasma XOD levels in humans with NAFLD in comparison to healthy subjects; an increase in its activity has only been found in the serum of rats with NAFLD [53].

However, patients with a high IFC showed higher levels of the proinflammatory cytokine IL-6, which is in accordance with a previous study that evidenced higher pro-inflammatory cytokine levels (TNFα and IL-6) in NAFLD patients [54]. A progressive increase in IL-6 was also found in steatosis and NASH patients [55], as well as in patients with metabolic syndrome [56]. IL-6 has also been related to hepatocellular carcinoma (HCC), the most common liver cancer, mainly in males, both in humans and in mice, probably due to the inhibitory effects of oestrogens on IL-6 production in females [57,58]. However, the current findings showed higher IL-6 plasma levels in women just at IFC = 0 (nonpathological), but no differences with males when pathological IFC levels were obtained. The shortage of differences may be explained by the menopause stage of female participants which places them in an endocrinological situation similar to that of men, without the oestrogenic inhibition of IL-6 production.

CK-18, an inflammatory intermediate filament protein of hepatocytes, is released into the circulation when hepatocyte damage occurs, making it a biomarker of disease progression in NAFLD and liver injury [59]. Since CK18 is cleaved by caspases, the levels of CK18 in serum can be indicative of hepatocyte apoptosis, a typical feature of liver injury [60]. The current study revealed that CK-18 significantly increased with the liver steatosis. Previous studies described CK-18 as a noninvasive marker, which could allow for the identification of patients with NAFLD and it has also been reported a relation between CK-18 levels with the evolution of NAFLD [59,60].

Irisin, a cytokine secreted by muscles after physical exercise and amarker of insulin resistance or metabolic disease [61], showed high levels in when the IFC was high. Similar results were previously observed, showing a direct association between the plasma irisin concentration and BMI in obese and NAFLD patients [61,62,63]. A recent study showed that fibronectin type III domain-containing 5 (FNDC5), which by proteolytic cleavage produces soluble irisin, is elevated in NAFLD [64], suggesting that FNDC5 increase in hepatocytes may be a mechanism to cushion the development of NAFLD by reducing hepatocyte steatogenesis and damage.

Resolvin D1, a lipid mediator involved in restoration of normal cellular function following the inflammation after tissue injury, as in obesity [65], showed high levels in participants with a high IFC. Its inverse was also significantly associated to pathological IFC. These resolvin levels may be related to a loss of the ability to respond to chronic subclinical inflammation, which would favour the progression of NAFLD. A multivariate logistic regression analysis applied in the current study showed a direct association between the inverse of resolvin D1 levels and IFC, showing that it may be a good IFC predictor.

FGF21 is a protein hepatokine mainly released from hepatocytes [66], and previous studies reported that FGF21 levels increased in NAFLD patients [67,68]. In a 3-year prospective population-based cohort, FGF21 levels were elevated in patients who progressed to NAFLD when compared with patients who did not [69]. Then it has been pointed out that the serum FGF21 level was a good biomarker for NAFLD diagnosis [66]. However, the current findings did not evidence any changes in FGF21 levels as the IFC increased. It may be explained since all participants were obese and have metabolic syndrome features; therefore they may have elevated levels in all the groups studied. It has also been pointed out that increased FGF21 circulating levels in over-nutrition could show the presence of compensatory responses by FGF21 to the underlying metabolic stress [70]. Additionally, FGF21 has direct anti-inflammatory and antifibrotic effects on the liver that are not associated with insulin resistance and obesity [71,72]. Thus, the absence of differences in this marker could also be due to the fact that patients only present liver steatosis and not steatohepatitis which implies inflammation and fibrosis.

Finally, the current findings from the ROC curve and area under the curve, as well as the multivariate logistic regression, showed that the plasma levels of CAT, SOD, CK-18, irisin, IL-6 and MDA, as well as the inverse of resolvin D1 levels, may be good IFC markers useful in clinical practice.

It has been also pointed out that NAFLD by itself is associated with cardiovascular events [73], and may precede and/or promote the development of T2DM, hypertension, and atherosclerosis/CVD in a bi-directional relationship between NAFLD and metabolic syndrome components, in particular T2DM and hypertension [74]. All these results pointed out that NAFLD is a systemic disease, and not just a hepatic disease [75]. The latest review also pointed out that NAFLD is linked to chronic kidney disease, as well as to endocrine, pulmonary, dermatological, gynaecological and haematological disorders, and to several cancers [76], emphasizing that HAFLD is more than just a disease.

Our previous findings showed that a higher dietary inflammatory index was associated with a high degree of liver damage in obese, with relevant noninvasive liver markers (ALT, AST, GGT) and with fatty liver index (FLI) [32]. BMI and metabolic syndrome have been also associated with oxidative stress (MDA, MPO, CAT) and pro-inflammatory markers (IL-6, high sensitivity C-reactive protein) [54,77,78]. The plasma antioxidant enzymatic activities were low and oxidative damage markers were high in patients at high cardiovascular risk [79].

Conversely, it can be hypothesized that the increased oxidative stress and proinflammatory environment appeared both in NAFLD, CVD, T2DM, and metabolic syndrome, perhaps follows the same initial stimulus (high dietary intake, low physical activity and high fat deposition in adipocytes [80]), and reinforce each other, joining a local and a systemic response. In this way, obesity, metabolic syndrome, hyperlipidaemia, atherosclerosis, and thrombosis were previously related with oxidative stress and a low-grade inflammation status [81,82,83,84]. In any case, this hypothesis needs further research.

## 5. Strengths and Limitations

The main strength of the current study is the association between oxidative stress and proinflammatory biomarker plasma levels and IFC, which may be also useful for diagnostic purposes in clinical practices. A limitation of this study is that no liver biopsies have been taken. However, IFC assessments have been made using MRI, which is a well-accepted, reliable, and noninvasive technique to do it, significantly reducing the risk for the patients. A second limitation is that sample size was relatively small. However, this sample size was enough to demonstrate the differences in the biomarker levels between IFCs. A third limitation may be inter-observer variations in anthropometric measurements. In order to avoid this, an accurate training of personnel has been done.

## 6. Conclusions

The current study has shown that as the intrahepatic fat content progresses, the markers of oxidative stress, plasma proinflammatory status, and CK18 significantly increase in patients according to the IFC diagnosed with MRI. Because diagnostic tests such as MRI are not routinely performed in clinical practices to diagnose or monitor fatty liver disease, combining various noninvasive markers would allow for the monitoring and evolution of NAFLD.

## Figures and Tables

**Table 1:** Characteristics of participants with nonalcoholic fatty liver disease (NAFLD) according to the intrahepatic fat content (IFC).

Sociodemographic and Clinical Characteristics	Reference Values	IFC = 0 (*n* = 30)	IFC = 1 (*n* =35)	IFC ≥ 2 (*n* = 35)	*p*-Value
Age (years)		52.5 ± 1.1	53.5 ± 1.2	51.7 ± 1.1	0.510
Female [*n* (%)]		16 (54.5)	17 (48.6)	14 (40.7)	0.136
Male [*n* (%)]		14 (45.5)	18 (50.0)	21 (59.3)	
Currently smoking [*n* (%)]		3 (9.3)	4 (12.5)	1 (3.7)	0.138
Currently alcohol consumption [*n* (%)]		5 (16.3)	6 (17.5)	4 (11.1)	0.912
Hypoglycaemic drugs [*n* (%)]		7 (23.1)	8 (23.7)	10 (29.6)	0.869
Antihypertensive drugs [*n* (%)]		14 (46.2)	15 (42.1)	16 (44.4)	0.507
Lipid-lowering drugs [*n* (%)]		14 (46.2)	9 (26.3)	13 (37.0)	0.231
Other drugs [*n* (%)]		21 (69.2)	24 (68.4)	22 (63.0)	0.507
Weight (kg)		88.0 ± 2.5	94.2 ± 2.5	95.1 ± 1.9	0.073
Height (cm)		164.5 ± 1.5	168.8 ± 1.7	166.2 ± 1.6	0.165
BMI (kg/m^2^)		32.4 ± 0.7	33.0 ± 0.7	34.3 ± 0.6	0.109
Systolic blood pressure (mmHg)	<130	131.3 ± 2.0	134.4 ± 1.9	140.9 ± 1.9 *	0.003
Diastolic blood pressure (mmHg)	<85	74.3 ± 1.1	80.7 ± 0.9 *	84.3 ± 1.1 * #	<0.001
Glucose (mg/dL)	70–110	99.9 ± 3.4	109.3 ± 3.6	121.7 ± 6.2 *	0.005
Hb1Ac (%)	3.8–6.2	5.68 ± 0.09	5.80 ± 0.15	7.14 ± 0.65 * #	0.015
Triglycerides (mg/dL)	<149	132.1 ± 8.6	207.6 ± 19.5	254.5 ± 42.4 *	0.010
HDL-cholesterol (mg/dL)	≥60	51.0 ± 1.3	42.8 ± 1.1 *	41.3 ± 0.9 *	<0.001
LDL-cholesterol (mg/dL)	<100	142.5 ± 3.5	126.2 ± 3.6 *	133.5 ± 3.9	0.007
Cholesterol total (mg/dL)	<200	219.7 ± 4.2	209.7 ± 5.1	221.4 ± 6.7	0.254
Bilirubin (mg/dL)	0.2–1.2	0.703 ± 0.337	0.713 ± 0.427	0.654 ± 0.047	0.571
AST (U/L)	5–34	22.6 ± 1.8	25.0 ± 1.7	28.9 ± 1.8 *	0.045
ALT (U/L)	0–55	27.8 ± 2.2	33.2 ± 4.4	45.2 ± 3.1 * #	0.002
GGT (U/L)	12–64	48.5 ± 5.9	48.2 ± 5.4	49.5 ± 5.2	0.983
CRP (mg/dL)	0.0–0.5	0.438 ± 0.073	0.523 ± 0.068	0.552 ± 0.074	0.524
Haematocrit (%)	40.0–50.0	43.6 ± 0.4	44.4 ± 0.3	43.7 ± 0.5	0.243
Erythrocytes (10^6^/μL)	4.50–5.80	4.86 ± 0.05	5.00 ± 0.04	4.89 ± 0.05	0.088
Leukocytes (10^3^/μL)	4.00–11.00	7.06 ± 0.20	7.46 ± 0.18	7.31 ± 0.23	0.377
Platelets (10^3^/μL)	150.0–400.0	232.9 ± 6.2	229.8 ± 4.5	245.1 ± 6.0	0.117

Values are the mean ± SEM except for male vs. female IFC, currently smoking, alcohol consumption and drug intake [*n*(%)]. Abbreviations: NAFLD, nonalcoholic fatty liver disease; BMI, body mass index; Hb1Ac, glycated haemoglobin 1A; HDL-cholesterol, high density lipoprotein; LDL-cholesterol, low density lipoprotein; AST, aspartate aminotransferase; ALT, alanine aminotransferase; GGT, gamma glutamyl transferase; CRP, c-reactive protein. Cross-validated estimates of the diagnostic accuracy of MRI-PDFF threshold for grading hepatic steatosis were: IFC = 0 (<6.4%), IFC = 1 (6.4–17.39%), and IFC ≥ 2 (>17.4%). One-way ANOVA (*p* < 0.05): * respect to IFC = 0; # respect to IFC = 1. Differences of male vs. female IFC, currently smoking, alcohol consumption and drug intake were analysed by χ^2^.

**Table 2:** Oxidative stress and inflammatory markers in the plasma of patients with NAFLD according to the intrahepatic fat content (IFC).

	IFC = 0 (*n* = 30)	IFC = 1 (*n* = 35)	IFC ≥ 2 (*n* = 35)	*p*-Value
	(Mean ± SEM)	(Mean ± SEM)	(Mean ± SEM)	
**Enzymatic Activities**				
CAT (kat/L sang)	28.3 ± 0.8	39.4 ± 0.9 *	60.0 ± 1.7 * #	<0.001
Women	31.8 ± 1.2	51.9 ± 4.3	60.8 ± 1.0	
Men	29.9 ± 1.6	42.1 ± 1.6	50.3 ± 2.1	
*p*-value †	0.355	0.015	0.002	
SOD (pkat/L sang)	277 ± 9	271 ± 9	307 ± 8 * #	0.011
Women	281 ± 9	269 ± 7	330 ± 26	
Men	261 ± 13	294 ± 14	257 ± 32	
*p*-value †	0.214	0.218	0.182	
**ELISA Assays**				
MPO (ng/mL)	5.55 ± 0.52	5.01 ± 0.37	5.12 ± 0.61	0.708
Women	4.77 ± 0.45	5.29 ± 0.66	7.12 ± 2.26	
Men	6.09 ± 0.84	4.55 ± 0.38	7.91 ± 1.46	
*p*-value †	0.151	0.302	0.777	
XOD (ng/mL)	0.398 ± 0.017	0.382 ± 0.014	0.396 ± 0.021	0.747
Women	0.378 ± 0.021	0.429 ± 0.037	0.323 ± 0.048	
Men	0.395 ± 0.021	0.395 ± 0.020	0.372 ± 0.048	
*p*-value †	0.566	0.379	0.513	
Irisin (ng/mL)	60.7 ± 10.2	92.6 ± 15.3	107.2 ± 12.7 *	0.041
Women	74.8 ± 17.8	103.1 ± 22.6	129.1 ± 51.0	
Men	55.9 ± 11.2	95.2 ± 16.9	99.8 ± 41.4	
*p*-value †	0.387	0.781	0.286	
IL-6 (pg/mL)	10.5 ± 1.1	14.4 ± 2.3	21.3 ± 4.6 *	0.041
Women	13.3 ± 0.8	15.4 ± 2.2	22.1 ± 4.2	
Men	6.7 ± 0.6	16.1 ± 2.9	9.6 ± 1.3	
*p*-value †	0.001	0.870	0.094	
Resolvin D1 (pg/mL)	157.5 ± 5.4	142.8 ± 4.6	137.9 ± 4.2 *	0.011
Women	154.5 ± 32.6	132.7 ± 8.6	102.0 ± 26.1	
Men	157.9 ± 8.5	154.0 ± 6.3	139.2 ± 14.0	
*p*-value †	0.727	0.053	0.189	
**Oxidative Damage**				
MDA (nM)	1.05 ± 0.09	1.95 ± 0.10 *	2.04 ± 0.11 *	<0.001
Women	0.94 ± 0.08	2.03 ± 0.13	1.47 ± 0.10	
Men	1.32 ± 0.13	1.67 ± 0.10	2.38 ± 0.30	
*p*-value †	0.016	0.080	0.038	
Protein carbonyl (%)	30.1 ± 3.4	34.1 ± 3.7	35.3 ± 3.0	0.614
Women	29.2 ± 4.5	38.8 ± 6.1	24.7 ± 6.2	
Men	31.3 ± 3.9	30.6 ± 3.0	19.8 ± 2.8	
*p*-value †	0.742	0.219	0.410	

Abbreviations: CAT, Catalase; SOD, Superoxide dismutase. MPO, Myeloperoxidase; XOD, Xanthine Oxidase; IL-6, Interleukin 6. MDA, Malondialdehyde. SEM, Standard Error of the Mean. Cross-validated estimates of diagnostic accuracy of the MRI-PDFF threshold for grading hepatic steatosis were: IFC = 0 (<6.4%), IFC = 1 (6.4–17.39%), IFC ≥ 2 (>17.4%). One-way ANOVA (*p* < 0.05): * respect to IFC = 0; # respect to IFC = 1; † women vs. men.

**Table 3:** Association of biomarkers and intrahepatic fat content (IFC).

Biomarkers	IFC = 0	IFC ≥1	*p*-Value
Catalase	1.00 (ref.)	1.208 (1.138–1.281)	<0.001
Malondialdehyde	1.00 (ref.)	4.013 (3.693–5.320)	<0.001
Cytokeratin 18	1.00 (ref.)	1.030 (1.017–1.044)	<0.001
Superoxide dismutase	1.00 (ref.)	1.004 (1.000–1.009)	0.046
Irisin	1.00 (ref.)	1.004 (0.001–0.008)	0.024
Interleukin-6	1.00 (ref.)	1.037 (1.003–1.071)	0.031
Xanthine oxidase	1.00 (ref.)	0.593 (0.213–3.909)	0.650
Protein carbonyl	1.00 (ref.)	1.007 (0.991–1.024)	0.401
Myeloperoxidase	1.00 (ref.)	0.985 (0.915–1.060)	0.688
Fibroblast Growth Factor 21	1.00 (ref.)	0.998 (0.985–1.011)	0.732
1/Resolvin D1	1.00 (ref.)	1.445 (1.347–1.602)	0.012

IFC = 0 (nonpathological) as reference value as reference value and IFC ≥ 1 (pathological) (as the dependent variable) and biomarkers after adjustment for sex, smoking, and alcohol consumption (categorical variables), and age, (continuous variable). CI: Coefficient Interval. ref.: reference.
